# Implication of the oligomeric state of the N-terminal PTX3 domain in cumulus matrix assembly

**DOI:** 10.1016/j.matbio.2011.05.002

**Published:** 2011-06

**Authors:** Elena Ievoli, Ragnar Lindstedt, Antonio Inforzato, Antonella Camaioni, Francesca Palone, Anthony J. Day, Alberto Mantovani, Giovanni Salvatori, Antonietta Salustri

**Affiliations:** a Department of Public Health and Cell Biology, University of Rome Tor Vergata, 00133 Rome, Italy; b Sigma-Tau Industrie Farmaceutiche Riunite S.p.A., 00040 Pomezia (RM), Italy; c Istituto Clinico Humanitas IRCCS, via Manzoni 113, 20089 Rozzano, Italy; d Wellcome Trust Centre for Cell-Matrix Research, Faculty of Life Sciences, University of Manchester, Oxford Road, Manchester M13 9PT, United Kingdom; e Department of Translational Medicine, University of Milan, Rozzano, 20089, Italy

**Keywords:** PTX3, Pentraxin 3, N___PTX3, N-terminal region of PTX3, HA, hyaluronan, IαI, inter-α-trypsin inhibitor, HCs, heavy chains of IαI, TSG-6, tumor necrosis factor-induced protein 6, FGF2, fibroblast growth factor 2, COC, cumulus cell oocyte complex, FSH, follicle-stimulating hormone, FBS, fetal bovine serum, Pentraxin3, Cumulus matrix assembly, Hyaluronan, IαI, TSG-6, FGF2

## Abstract

Pentraxin 3 (PTX3) plays a key role in the formation of the hyaluronan-rich matrix of the cumulus oophorus surrounding ovulated eggs that is required for successful fertilization and female fertility. PTX3 is a multimeric protein consisting of eight identical protomers held together by a combination of non-covalent interactions and disulfide bonds. Recent findings suggest that the oligomeric status of PTX3 is important for stabilizing the cumulus matrix. Because the role of PTX3 in the cumulus resides in the unique N-terminal sequence of the protomer, we investigated further this issue by testing the ability of distinct Cys/Ser mutants of recombinant N-terminal region of PTX3 (N___PTX3) with different oligomeric arrangement to promote *in vitro* normal expansion in cumuli from *Ptx3*-null mice. Here we report that the dimer of the N___PTX3 is unable to rescue cumulus matrix organization, and that the tetrameric assembly of the protein is the minimal oligomeric state required for accomplishing this function. We have previously demonstrated that PTX3 binds to HCs of IαI and TSG-6, which are essential for cumulus matrix formation and able to interact with hyaluronan. Interestingly, here we show by solid-phase binding experiments that the dimer of the N___PTX3 retains the ability to bind to both IαI and TSG-6, suggesting that the octameric structure of PTX3 provides multiple binding sites for each of these ligands. These findings support the hypothesis that PTX3 contributes to cumulus matrix organization by cross-linking HA polymers through interactions with multiple HCs of IαI and/or TSG-6. The N-terminal PTX3 tetrameric oligomerization was recently reported to be also required for recognition and inhibition of FGF2. Given that this growth factor has been detected in the mammalian preovulatory follicle, we wondered whether FGF2 negatively influences cumulus expansion and PTX3 may also serve *in vivo* to antagonize its activity. We found that a molar excess of FGF2, above PTX3 binding capacity, does not affect *in vitro* cumulus matrix formation thus ruling out this possibility. In conclusion, the data strength the view that PTX3 acts as a nodal molecule in cross-linking HA in the matrix.

## 1 Introduction

Pentraxin 3 (PTX3) is a homo-octameric secreted glycoprotein composed of 42 kDa subunits that belongs to the long-pentraxin family (Inforzato et al., 2008, 2010). The amino acid sequence of each protomer is highly conserved from mouse to humans and consists of two structural domains: a C-terminal 203-amino acid region sharing homology with the classical short pentraxins C-reactive protein and serum amyloid P component, and a unique N-terminal domain of 178 amino acids that does not show homology with any other known protein (Breviario et al., 1992). PTX3 is produced and released by a broad variety of cells and tissues in response to inflammatory stimuli and shows multifunctional properties likely due to its ability to interact with a number of different ligands. PTX3 plays a non-redundant role *in vivo* in recognition of and innate resistance to selected microorganisms and it modulates inflammatory reactions and angiogenesis (Garlanda et al., 2002; Presta et al., 2007; Bottazzi et al., 2009). Furthermore, *Ptx3*-null mice show a severe defect in female fertility (Varani et al., 2002). PTX3 is synthesized, under ovulatory stimuli, in the ovarian preovulatory follicle by mouse and human cumulus cells surrounding the oocyte (i.e., the cumulus oophorus or cumulus cell oocyte complex, COC), and localizes in the cumulus extracellular matrix (Salustri et al., 2004). The major component of this newly synthesized matrix is hyaluronan (HA), which is responsible for the viscoelastic properties and volumetric expansion of cumulus oophorus (Russell and Salustri, 2006). Female mice lacking *Ptx3* do not correctly assemble the cumulus matrix and consequently oocytes are not fertilized. *In vitro* hormone stimulation of *Ptx3*^*−/−*^ COCs showed that cumulus cells synthesize HA at a normal rate but are unable to organize this polymer in a matrix unless recombinant PTX3 is added to the culture medium (Salustri et al., 2004). Studies performed with recombinant preparations of both N- and C-terminal domains of PTX3 have shown that this function is exerted exclusively by the N-terminal region (Scarchilli et al., 2007). Amongst the known PTX3 ligands, only the heavy chains (HCs) of inter-α-trypsin inhibitor (IαI) (Scarchilli et al., 2007) and FGF2 (Camozzi et al., 2006) have shown the capacity to interact exclusively with this portion of the molecule.

It is well documented that PTX3/FGF2 interaction prevents the binding of FGF2 to its cognate receptors, thereby inhibiting its activity (Rusnati et al., 2004; Camozzi et al., 2005). FGF2 is involved in numerous cellular functions in various cell types including matrix remodeling, i.e. this growth factor appears to exert catabolic and anti-anabolic effects on both human articular cartilage and intervertebral disc matrix (Ellman et al., 2008). FGF2 mRNA transcript and protein have been detected in mammalian preovulatory follicles (Guthridge et al., 1992; Di Blasio et al., 1993; Seli et al., 1998; Berisha et al., 2004; Hammadeh et al., 2004), but whether FGF2 affects cumulus expansion and its neutralization by PTX3 within the COC is required for correct matrix formation has not been investigated yet.

Conversely, several lines of evidence both *in vitro* and *in vivo* suggest that the interaction of PTX3 with HCs of IαI is important in the organization of HA strands in the gel-like matrix of the cumulus. IαI is a serum component consisting of two homologous HC proteins covalently linked to the chondroitin sulfate moiety of bikunin (Salier et al., 1996). IαI diffuses into the ovarian follicle and HCs are transferred onto HA synthesized by cumulus cells during cumulus expansion. Covalent linkage of HCs to HA is essential, in addition to PTX3, for cumulus matrix assembly (Zhuo et al., 2001; Fulop et al., 2003). Cooperation of PTX3 and HCs in matrix assembly and stability is suggested by the observations that the two molecules associate within the matrix and cumulus expansion is inhibited by a PTX3 antibody that blocks their interaction (Scarchilli et al., 2007). PTX3 has also the capacity to interact *in vitro* with TSG-6 (Salustri et al., 2004), a protein synthesized by cumulus cells (Mukhopadhyay et al., 2001) with the ability to bind non-covalently to HA (Lee et al., 1992; Kohda et al., 1996). Based on these findings, we have proposed that PTX3 provides the binding for multiple HCs of IαI (Scarchilli et al., 2007), and possibly for TSG-6 (Salustri et al., 2004), thereby cross-linking HA strands. This hypothesis would imply that the multimeric organization of PTX3 is of relevance for matrix stability. Cysteine to serine (Cys/Ser) site-directed mutagenesis showed that, although PTX3 protomers have a certain ability to self-assemble into oligomers, three interchain disulfide bonds in the N-terminal domain and two in the C-terminal region support the octameric assembly of the molecule (Inforzato et al., 2008). Interestingly, the PTX3 mutant lacking the entire network of inter-chain disulfide bonds, which mainly forms dimers, was less effective than other mutants, prevalently composed of tetramers and octamers, in restoring cumulus expansion in *Ptx3* ^*−/−*^ COCs.

In the present study we investigated further the impact of PTX3 oligomerization on its biological function and ligand recognition by using distinct Cys/Ser mutants of the recombinant PTX3 N-terminal domain (N_PTX3). We demonstrate unambiguously that a tetrameric arrangement of the N-terminal portion of PTX3 is the minimal oligomeric organization of the protein required for supporting cumulus matrix formation. Findings are also presented indicating that N_PTX3 interacts with multiple HCs and TSG-6, further strengthening the view that PTX3 acts as a nodal molecule in cross-linking HA in the matrix. Finally, we obtained data suggesting that the antagonist activity of PTX3 on FGF2 is not required for cumulus matrix stability.

## 2 Results

### 2.1 Tetramerisation of N_PTX3 is required for cumulus matrix organization

The N-terminal domain of PTX3 contains three cysteine residues at positions 47, 49 and 103, which form interchain disulfide bonds stabilizing the protein octamer (Inforzato et al., 2008). In order to determine the minimum oligomeric structure of PTX3 needed for organizing the extracellular matrix of cumulus oophorus we used single (N_PTX3C103S), double (N_PTX3C47S/C49S) and triple (N_PTX3C47S/C49S/C103S) mutant constructs of the recombinant N_PTX3 obtained by the replacement of cysteines at amino acid positions 103, 47/49 and 47/49/103 with serine residues.

**Fig. 1:**
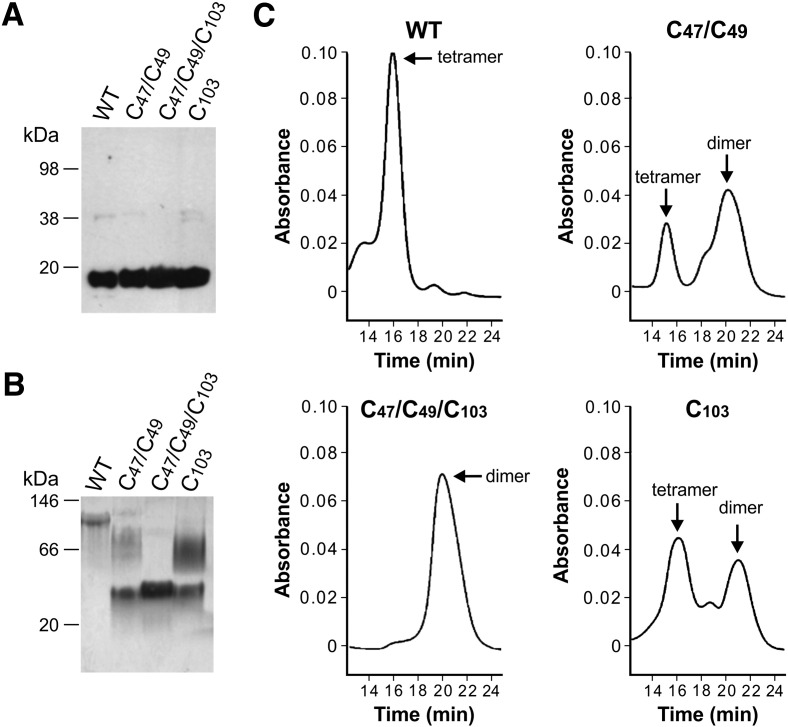
Oligomeric status of wild type N_PTX3 and its Cys/Ser mutants. Electrophoretic analysis under either (A) denaturing and reducing or (B) native conditions. A gel that is representative of two independent experiments is shown. (C) Representative UV chromatograms from size exclusion chromatography (SEC) analysis of wild type N_PTX3 (WT) and its single (C103), double (C47/C49) and triple (C47/C49/C103) Cys/Ser mutants. Peaks corresponding to tetramers and dimers (reference to Inforzato et al., 2010) are indicated.

As expected, all proteins migrated as a single band corresponding to the monomeric form (~ 18 kDa) on SDS-PAGE under reducing conditions (Fig. 1A). Consistent with recent investigations (Inforzato et al., 2010), analysis of the oligomeric state of these constructs in native conditions by size exclusion chromatography (Fig. 1C) and gel electrophoresis (Fig. 1B) showed that wild type N_PTX3 was organized into tetramers (~ 74 kDa) and that the single and double mutants were both composed of a mixture of tetramers (~68 kDa) and dimers (~33 kDa), with a preponderance of tetramers on dimers for the single mutant and the opposite for the double mutant, whereas the triple mutant formed an homogeneous species composed entirely of dimers.

**Fig. 2:**
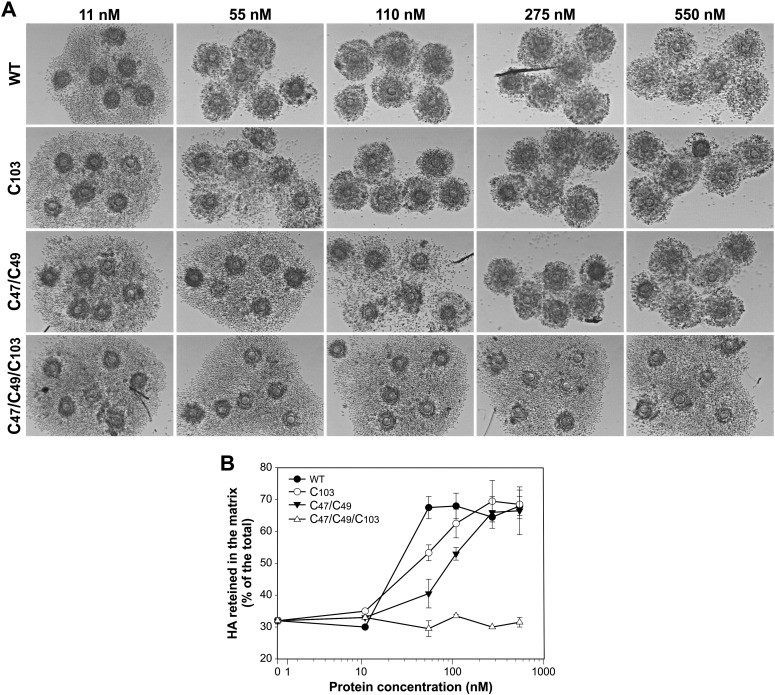
Ability of N_PTX3 Cys/ Ser mutants to promote cumulus expansion and HA organization in *Ptx3*^−/−^ COCs. (A) Micrographs showing the morphology of COCs from *Ptx3* null mice cultured for 16 h with 100 ng/ml FSH, 2% FBS in the presence of wild type N_PTX3 (WT) or N_PTX3 Cys to Ser single (C103), double (C47/C49) and triple (C47/C49/C103) mutants at different concentrations calculated based on the protomer molecular mass (i.e., ~18 kDa). (B) The corresponding amounts of newly synthesized HA retained in the matrix and released into the medium were determined. The reported values represent the mean ± S.D. of the percentage of HA retained in the matrix, of the total synthesized, obtained from three independent experiments.

The ability of these mutant proteins to promote HA organization in the viscoelastic cumulus matrix was investigated by *in vitro* expansion of *Ptx3* deficient COCs. Cumuli were isolated from *Ptx3*^−/−^ mice and stimulated with follicle stimulating hormone (FSH) in the presence of increasing concentrations of wild type recombinant N_PTX3 and the Cys/Ser N_PTX3 mutants. Radiolabeled precursors of glycosaminoglycans were added to the culture for metabolic labeling of the newly synthesized HA. COC morphology (Fig. 2A) and HA distribution between the culture medium and matrix (Fig. 2B) were evaluated after 16 h of hormone treatment. At the minimum applied dose of 11 nM, all the proteins were ineffective to support matrix organization, as shown by both the release of HA in the medium and the dispersion of cumulus cells. The single mutant, forming more tetramers than dimers, was slightly less effective than the wild type N_PTX3, that was assembled exclusively as tetramers, allowing maximum HA retention in the matrix and COC integrity at the concentration of 110 nM instead of 55 nM. A lower activity was shown by the double mutant, organized more in dimers than in tetramers, which restored the normal phenotype only at a dose of 275 nM. The triple mutant, lacking the entire set of disulfide bonds and composed entirely of non-covalent dimers, was unable to promote HA organization in the matrix and normal cumulus expansion even at a dose 10 times higher than minimum effective dose of wild type N_PTX3. These results indicate that the organization of N_PTX3 in tetramers is necessary to support cumulus matrix formation.

### 2.2 N_PTX3 contains multiple binding sites for IαI and TSG-6

**Fig. 3:**
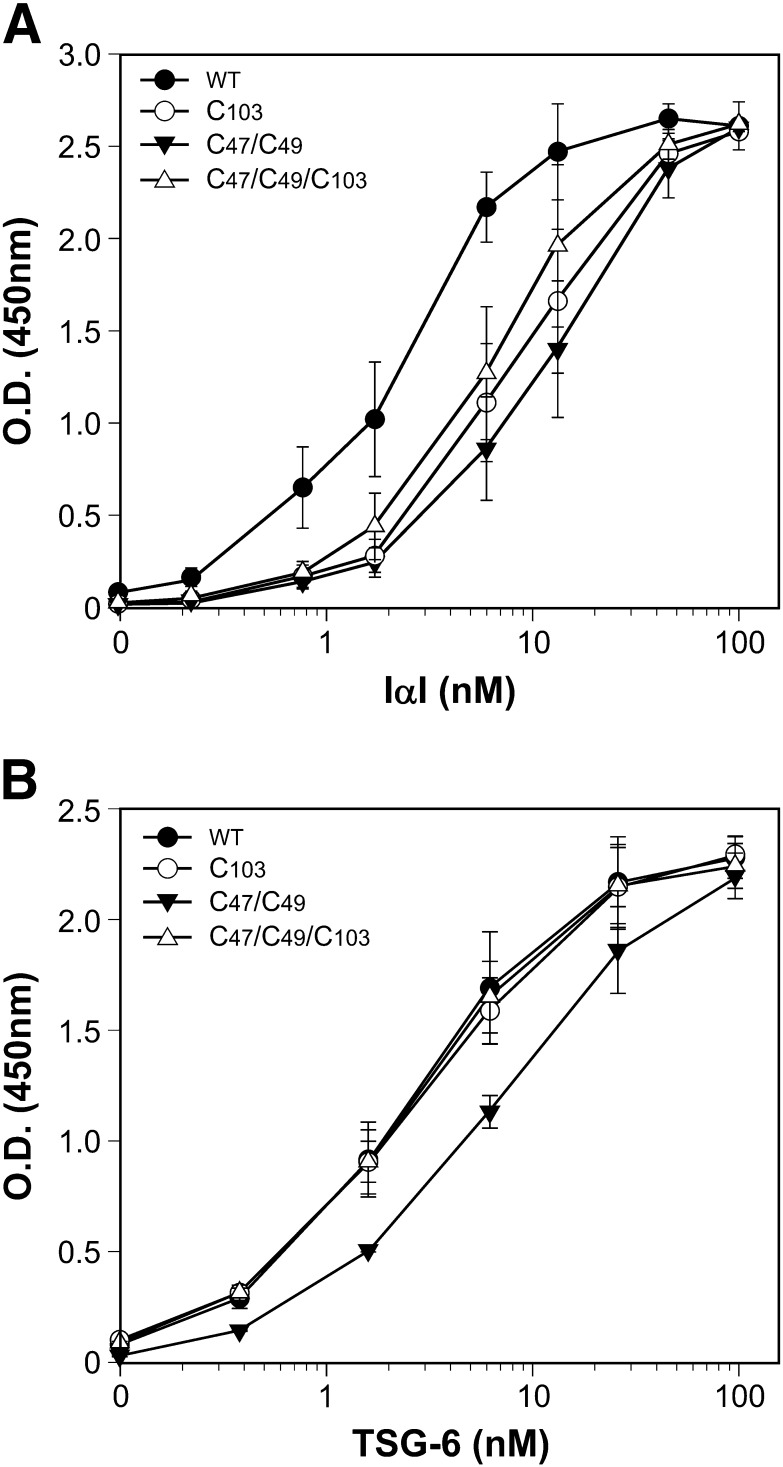
Binding of the N_PTX3 mutants to IαI and TSG-6. Dose-response binding of different amounts of IαI (A) or TSG-6 (B) to immobilized wild type N_PTX3 (WT) and its single (C103), double (C47/C49), and triple (C47/C49/C103) mutants. All the assays were performed at pH 7.4 in the presence of PBS with Ca^2+^ and Mg^2+^. The extent of binding was determined by enzyme-linked immunosorbent assay. Values of four independent experiments done in duplicate are plotted (mean ± S.D.).

**Fig. 4:**
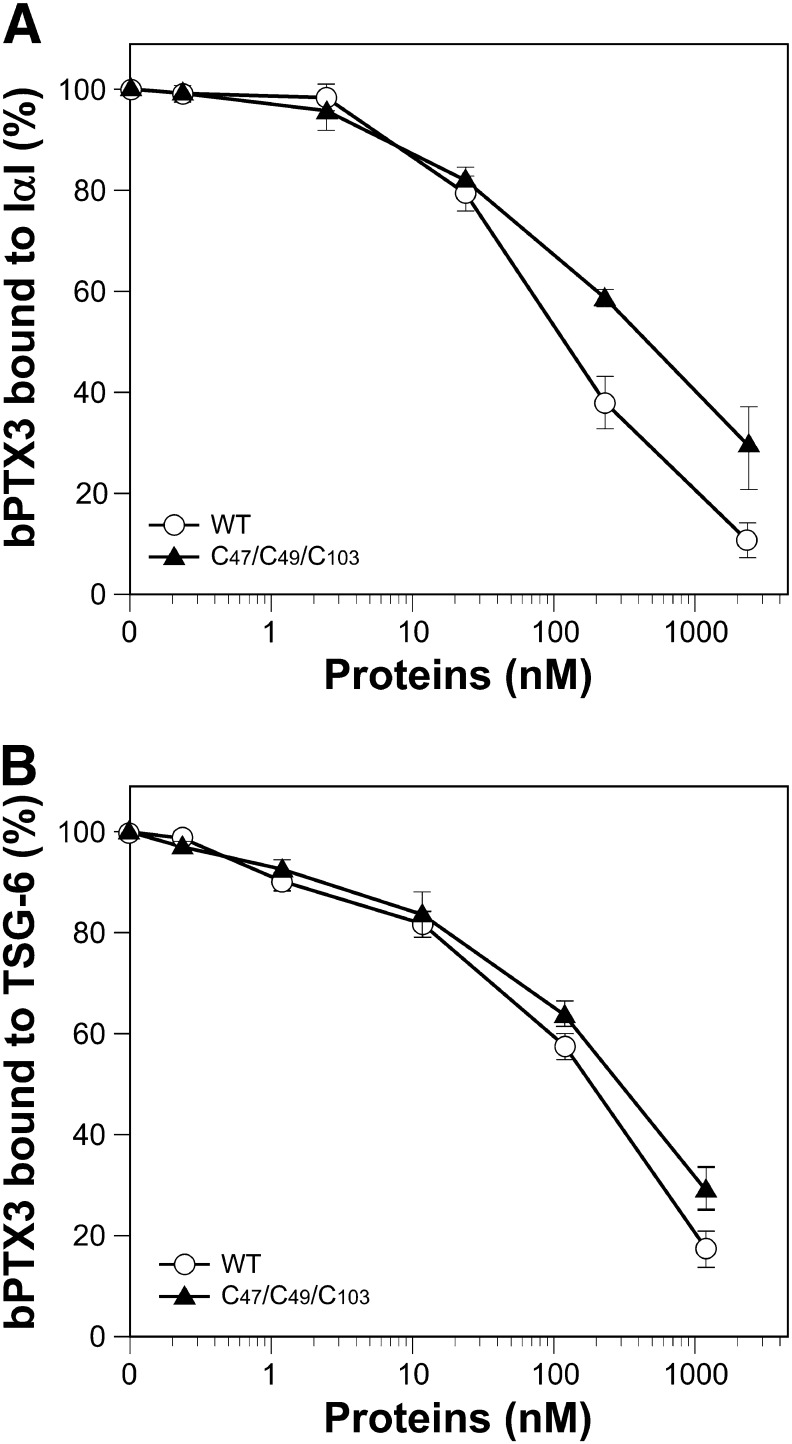
Competition of the biotinylated PTX3 binding to IαI and TSG-6 by N_PTX3 and its N_PTX3C47S/C49S/C103S triple mutant. The binding of biotinylated PTX3 (bPTX3) to plate wells coated with either IαI (A) or TSG-6 (B) was assessed in the presence of either N_PTX3 (WT) or N_PTX3C47S/C49S/C103S (C47/C49/C103) at different concentrations. Bound bPTX3 was detected with HRP-conjugated streptavidin. Data from two independent experiments (mean ± S.D.) are expressed as percentage of bPTX3 binding in the absence of competitors.

We have previously proposed that PTX3 participates in cumulus matrix organization by interacting with multiple molecules of HCs of IαI and/or of TSG-6, thus cross-linking distinct HA strands (Salustri et al., 2004; Scarchilli et al., 2007). Based on this hypothesis, the tetramers of the recombinant N_PTX3 protein should contain at least two binding sites for these ligands in order to perform this function. Therefore, we tested whether dimers of N_PTX3 were sufficient to support the binding of IαI and TSG-6 by microtiter plate-based binding assays. Results reported in Fig. 3A show that IαI interacted with the immobilized N_PTX3 proteins in a dose-dependent manner, where all of the mutants bound to a similar extent, regardless of their tetrameric or dimeric organization, albeit with a somewhat lower binding affinity than to wild type protein. Moreover, results presented in Fig. 3B show for the first time that TSG-6, as in the case for the IαI, recognized the N-terminal domain of PTX3, and that both the single and the triple mutants of N_PTX3 had a TSG-6 binding activity identical to the wild type N_PTX3, with a small difference for the double mutant. Accordingly, the triple mutant of N_PTX3, that only forms dimers, could compete for the binding of biotinylated recombinant full length PTX3 to either IαI or TSG-6 with a potency comparable to that of wild type N_PTX3 (Fig. 4A and B). Therefore, dimers of N_PTX3 retain the ability to bind to both IαI and TSG-6. These findings are consistent with the hypothesis that in the cumulus matrix the octameric full length PTX3 contributes to HA cross-linking by establishing multiple interactions with these HA-bound proteins.

### 2.3 FGF2 antagonizing activity of PTX3 is not involved in cumulus expansion

**Fig. 5:**
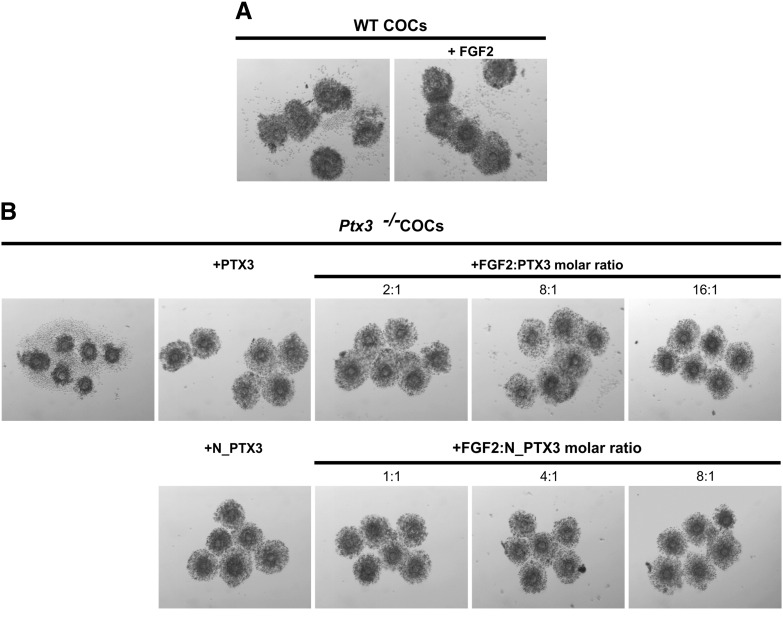
Effect of exogenous FGF2 on cumulus expansion. Micrographs showing the morphology of (A) wild type COCs cultured for 16 h with 100 ng/ml FSH, 2% FBS in the absence and in the presence of 100 ng/ml FGF2; (B) *Ptx3* null COCs cultured with 100 ng/ml FSH, 2% FBS without or with either 3 nM full length recombinant PTX3 (Mw = ~ 340 kDa) or 14 nM recombinant N_PTX3 (Mw = ~ 70 kDa) in the presence of FGF2 (Mw = ~ 17.5 kDa).

It has been recently shown that PTX3 contains two binding sites for FGF2, each formed by a tetramer of the N-terminal domain (Inforzato et al., 2010). Given that PTX3 has the ability to antagonize FGF2 action (Camozzi et al., 2005) and that this growth factor is synthesized by several cellular components of the mammalian follicle and secreted into the follicular fluid filling the follicle cavity (Guthridge et al., 1992; Di Blasio et al., 1993; Seli et al., 1998; Berisha et al., 2004; Hammadeh et al., 2004), we wondered whether FGF2 negatively affects COC expansion and PTX3 also serves *in vivo* to locally inactivate FGF2, in addition to directly participating in the formation of the matrix. If this were the case, then it would be expected that a molar excess of FGF2 over PTX3 would impair cumulus expansion. However, we observed that wild type COCs stimulated *in vitro* with FSH underwent normal expansion when the medium was supplemented with 100 ng/ml FGF2, a concentration much higher than that found in follicular fluid (about 100–200 pg/ml) (Seli et al., 1998; Hammadeh et al., 2004) (Fig. 5A). Furthermore, both full length PTX3 and N_PTX3 restored a normal phenotype in FSH-stimulated *Ptx3*^*−/−*^ COCs in the presence of over saturating concentrations of FGF2 over PTX3, based on the number of FGF2-binding sites in the PTX3 and N_PTX3 proteins (i.e., two and one, respectively; Inforzato et al., 2010) (Fig. 5B). These results indicate that FGF2 does not have an inhibitory effect on cumulus expansion, thereby ruling out a physiological relevance of FGF2/PTX3 interaction in this process.

## 3 Discussion

PTX3 plays a non-redundant role in the formation of cumulus extracellular matrix, which constitutes a suitable environment for sperm penetration and oocyte fertilization (Varani et al., 2002; Salustri et al., 2004).

This molecule is mainly composed of covalently linked octamers. The network of disulfide bonds supporting the octameric assembly was recently resolved (Inforzato et al., 2008, 2010). Cysteine residues at positions 47, 49, and 103 in the tetrameric N-terminal domain form three interchain disulfide bonds linking the protomers. Additional interchain disulfide bonds formed by the C-terminal domain cysteines at positions 317 and 318 are responsible for linking PTX3 tetramers into octamers. PTX3 site-directed mutagenesis of cysteines suggested that multimeric PTX3 organization is important for cumulus matrix assembly (Inforzato et al., 2008). Mutants exhibiting mainly octameric and tetrameric arrangement were as effective as wild type PTX3 in restoring normal phenotype in *Ptx3*^*−/−*^ COCs cultured *in vitro*. In contrast, the mutant lacking the entire network of interchain disulphide bonds and composed mainly of dimers and to a minor extent of octamers and tetramers, was less effective than the wild type protein, suggesting that the minimum PTX3 oligomeric status required for matrix stability is a tetramer. This hypothesis is corroborated by the findings reported in the present paper. We found that the recombinant N_PTX3 protein, which is effective as the full length PTX3 in cumulus matrix assembly, is composed of tetramers. In addition, a reduction in functionality was observed for the mutants N_PTX3C103S and N_PTX3C47S/C49S that correlates with their capacity to form dimers rather than tetramers. Consistent with this, the N_PTX3C47S/C49S/C103S mutant, composed only of dimers, was completely ineffective. The possibility that it is the Cys/Ser substitutions introduced in the primary sequence, rather than the protein's oligomeric state, that is affecting the activity of the N___PTX3 triple mutant C47S/C49S/C103S, is ruled out by the evidence that the full length PTX3 triple mutant C47S/C49S/C103S, which self-assembles into an octamer stabilized by disulphide bonds in the C-terminal domain, retains the ability to promote expansion in *Ptx3*^*−/−*^ COCs (Inforzato et al., 2008).

HCs are transferred from IαI to HA synthesized by cumulus cells during expansion by the catalytic action of TSG-6 (Fulop et al., 2003; Rugg et al., 2005), a process essential for cumulus matrix formation (Zhuo et al., 2001). PTX3 is not able to bind HA and does not take part in this process (Salustri et al., 2004), although it shows the ability to bind to IαI and TSG-6. The evidence that the bikunin component of IαI (i.e. the light chain and chondroitin sulfate chain without the heavy chains) neither binds to PTX3 nor competes for the binding of PTX3 to IαI suggests that PTX3 exclusively interacts with HCs of IαI, where they become an integral component of the cumulus matrix. A direct interaction and functional link between PTX3 and HCs *in vivo* is documented by their colocalization in the cumulus matrix, coimmunoprecipitation from cumulus matrix extracts and inhibition of cumulus expansion by a PTX3 antibody blocking their interaction. We have proposed that PTX3 binds multiple HCs covalently linked to HA, thereby acting as a node for cross-linking HA strands (Scarchilli et al., 2007). Binding of multiple molecules of the hyaladherin TSG-6 to PTX3 has also been suggested to strengthen the hyaluronan network (Salustri et al., 2004). Here we demonstrate for the first time that TSG-6, as IαI, binds to the N-terminal domain of PTX3, i.e. the portion of the molecule required and sufficient for organizing HA and for enabling matrix formation. In addition, consistent with the proposed model, we provide evidence here that PTX3 has indeed multivalent binding sites for HCs and TSG-6. The ability of IαI and TSG-6 to bind to the dimer of N_PTX3 (i.e., the N_PTX3 triple mutant) indicates that octameric full length PTX3 and tetrameric recombinant N_PTX3, both able to sustain matrix assembly, contain at least four and two binding sites for these ligands, respectively. The capacity of N_PTX3 to self-assembly into dimers in the absence of disulfide bonds prevented the possibility to assess whether individual subunits of N_PTX3 can interact with HCs or TSG-6. However, given the observation that the N_PTX3 triple mutant is unable to retain HA in the *Ptx3* deficient COC, it is likely that the dimers of the PTX3 N-terminal domain can support the binding of only a single molecule of either HC or TSG-6, a condition not sufficient to allow the cross-linking of HA strands.

Recent findings showed the requirement of the tetramer as the minimum PTX3 oligomeric state for FGF2 recognition (Inforzato et al., 2010), i.e. as we found here for cumulus matrix stabilization. The interaction with PTX3 inhibits FGF2 activity preventing the association to its receptor (Camozzi et al., 2005). This growth factor is synthesized by a variety of cells and plays a different role depending on the biological context. With regards to extracellular matrix homeostasis, recent evidence suggests that FGF2 exerts degradative effects on the hyaluronan-rich matrix of both human articular cartilage and intervertebral discs, via up regulation of matrix-degrading enzyme activity in these tissues (Ellman et al., 2008). *Fgf2* expression has been detected in rat, pig, cow and human theca and granulosa cells, which constitute the wall of the ovarian follicle (Guthridge et al., 1992; Di Blasio et al., 1993; Berisha et al., 2004), and FGF2 was found in human preovulatory follicular fluid (Seli et al., 1998; Hammadeh et al., 2004). It is noteworthy that FGF2 increases the expression of tissue type plasminogen activator in cultured granulosa cells (LaPolt et al., 1990), a proteolytic enzyme involved in extracellular matrix degradation and rupture of the follicle at the time of ovulation (Liu, 2004). All together these observations raised the possibility that sequestration of FGF2 by PTX3 in the cumulus might prevent the expression of matrix-degrading enzymes, thus providing an additional mechanism for cumulus matrix stabilization. However, our data do not support this hypothesis. We observed that FGF2 does not exert a negative effect on cumulus expansion. This growth factor did not alter the formation and the stability of the cumulus matrix *in vitro* when applied at higher concentrations than those found under physiological conditions. Analogously, normal expansion was restored in *Ptx3*^*−/−*^ COCs when cultured with either full length PTX3 or N_PTX3 in the presence of over saturating doses of FGF2.

In conclusion, these findings provide evidence that a tetramer is the functional unit of PTX3 that can support cumulus matrix formation, as previously hypothesized. Also, new insights are provided on the interaction of PTX3 with HCs of IαI and TSG-6 that further support the hypothesis of a cooperative action in cross-linking HA strands, thus conferring stability to the cumulus matrix.

## 4 Experimental procedures

### 4.1 Cloning, expression and purification of N-terminal PTX3 wild type and mutant proteins

The coding sequence of the N_PTX3 (residues 18–170 of the PTX3 preprotein; Bottazzi et al., 2009) was amplified by PCR from the plasmid vector pSG5hPTX3 containing the full-length human PTX3 cDNA sequence and subcloned into a pcDNA3.1/V5-His vector (Invitrogen). The 5′ primer, CTCGGTACCATGCATCTCCTTGCGATTCTG, contained a Kpn I restriction site and the 3′ primer, CTCACCGGTAGCCCAGCCCTGCAC, contained an Age I restriction site. The N_PTX3 cDNA coded for a protein with two non-authentic amino acids (Thr-Gly) and a 6-residue histidine tag at its C-terminal end. N_PTX3C103S, N_PTX3C47S/C49S, and N_PTX3C47S/C49S/C103S mutant constructs were made from the full length mutant expression vectors (Inforzato et al., 2008) by overlapping PCR using the same primers as for the N-terminal PTX3 wild type construct and subcloned into the pcDNA3.1/V5-His vector. The resulting expression plasmids (10 μg) were transfected into 1 × 10^7^ HEK-293 F cells (Invitrogen) suspended in GIBCO® FreeStyle™ 293 Expression Medium using Lipofectamine (Invitrogen) and stably transfected cells selected in expression medium containing 0.6 mg/ml G-418 (Invitrogen). Clarified cell culture supernatants were loaded onto 1-ml HisTrap Immobilized Metal Affinity Chromatography Columns (GE Healthcare Life Sciences AB, Uppsala, Sweden) and bound proteins eluted with phosphate buffered saline (PBS) supplemented with 120 mM imidazole. Recombinant protein containing fractions were pooled and desalted by gel filtration chromatography on 5-ml HiTrap desalting columns (GE Healthcare Life Sciences AB, Uppsala, Sweden), equilibrated and eluted with PBS. Purity of the recombinant protein was assessed by SDS-PAGE and was higher than 95%.

### 4.2 Size exclusion chromatography

Proteins were analyzed on a JASCO PU 1580 HPLC instrument connected to a Jasco UV-2077 Plus UV–visible spectrophotometer (Jasco, Easton, MD). Proteins were run on Superose 6, 300 × 10-mm gel filtration columns (GE Healthcare) equilibrated in 20 mM sodium phosphate, 150 mM NaCl, pH 7.4 (PBS) at 0.7 ml/min. Molecular weight standards (dextran blue, thyroglobulin, ferritin, catalase and bovine serum albumin) were purchased from GE Healthcare.

### 4.3 Gel electrophoresis

The purified proteins were denatured and reduced in Laemmli SDS-PAGE sample buffer containing 20 mM DTT and separated on precast Tris-glycine 4–20% (w/v) SDS-polyacrylamide gels (Invitrogen). To analyse the purified proteins under native conditions, protein aliquots were diluted in 20 mM Na_2_HP0_4_, pH 7.4 and separated on a precast 4–20% polyacrylamide gel in the absence of SDS. Protein bands were revealed using Simply Blue stain solution (Invitrogen).

### 4.4 Isolation and culture of cumuli

Adult Sv129 *Ptx3* deficient female mice, generated as described (Garlanda et al., 2002), were injected with 5 IU of pregnant mares' serum gonadotropin (PMSG; Folligon, Demas) and sacrificed 44–48 h later. Ovaries were removed and cumulus cell oocyte complexes (COCs) were mechanically isolated by puncturing large follicles in Eagle's minimum essential medium (MEM; GIBCO, Invitrogen) containing 25 mM HEPES (GIBCO, Invitrogen), 0.1% bovine serum albumine (BSA; Sigma), and 50 ng/ml gentamycin (GIBCO, Invitrogen). Compact COCs were cultured, under mineral oil (Sigma), in 20 μl droplets of MEM supplemented with 2% fetal bovine serum (FBS; GIBCO, Invitrogen), 3 mM glutamine (Sigma), 0.3 mM sodium pyruvate (Sigma) and 50 ng/ml gentamycin, in the presence of 100 ng/ml follicle stimulating hormone (highly purified rat-FSH; kindly provided by the NIDDK and the National Hormone and Pituitary Program, National Institutes of Health), at 37 °C with 5% CO_2_ in humidified air for 16 h. Human recombinant wild type PTX3, wild type or mutant N_PTX3 proteins and FGF2 (R&D) were added to the medium at the beginning of culture, at the concentrations indicated in the text.

### 4.5 Quantitation of HA

Cultures as described above were carried out in the presence of 100 μCi/ml [^3^H]-glucosamine hydrochloride (Perkin Elmer Life Sciences) for 16 h. At the end of culture, medium and cell matrix were collected separately and the amount of HA in the two compartments was assessed using a modification of the procedure described previously (Camaioni et al., 1993).

Briefly, each sample was treated with papain (50 μg/ml final concentration, Sigma-Aldrich) for 1 h at 65 °C. The extraction was completed by adding an equal volume of 8 M guanidine HCl containing 4% Triton X-100. All extracts were then eluted on a column of Sephadex G-50 (2 ml bed volume) equilibrated with 0.1 M Tris, 0.1 M sodium acetate and 0.5% Triton X-100, pH 7.3. The excluded volume was recovered and counted. Each sample was then digested with 1 IU of *Streptomyces* hyaluronidase (Calbiochem) for 2 h at 37 °C, and then chromatographed on a column of Sephadex G-50 (8 ml bed volume). The excluded and the included fractions were counted for radioactivity to determine the proportion of the radiolabeled macromolecules digested by the enzyme.

### 4.6 Solid-phase binding assay

Dose-response binding of IαI or TSG-6 to immobilized wild type or mutant N_PTX3 was assessed using colorimetric microtitre plate assays as follows. 96-well plates (Costar) were coated with 100 μl of wild type (N_PTX3), single (N_PTX3C103S), double (N_PTX3C47S/C49S) and triple (N_PTX3C47S/C49S/C103S) mutant N_PTX3 (10 μg/ml) in 15 mM NaHCO_3,_ 35 mM Na_2_CO_3_ buffer at pH 9.6 and incubated overnight at 4 °C. The plates were then washed three times with PBS with Ca^2+^ and Mg^2+^ (PBS^++^; GIBCO, Invitrogen), 0.05% Tween-20 (Sigma) (PBS^++^/T) and blocked for 2 h at 37 °C with PBS^++^/T containing 1% BSA. After three washes, purified human IαI (purified from human serum (Michalski et al. 1994); kindly supplied by Dr. Jacques Mizon, Université de Lille II, France) or mouse TSG-6 (R&D) was then added in a total volume of 100 μl diluted in PBS^++^/T, 1% BSA at the concentrations specified in the text and incubated for 2 h at 37 °C. The plates were washed five times with PBS^++^/T, and then incubated with 100 μl of rabbit polyclonal anti-human IαI antibody (1 μg/ml; DAKO) or 100 μl of goat polyclonal anti-mouse TSG-6 (1 μg/ml; R&D) in PBS^++^/T, 1% BSA at 37 °C for 1 h. After five washes with PBS^++^/T, plates were incubated with 100 μl of HRP-linked anti-rabbit F(ab′)_2_ fragment immunoglobulin (1:5000; GE Healthcare) or HRP-conjugated donkey anti-goat IgG (1:25000; Jackson ImmunoResearch) diluted in PBS^++^/T, 1% BSA for 1 h at 37 °C. The plates were washed five times with PBS^++^/T and the enzymatic reaction was developed using the substrate ditetra-methylbenzidine (TMB; Promega) for 10 min.

In competition experiments, plates were coated with 500 ng/well of TSG-6 or 500 ng/well of IαI and incubated with 50 ng/well of biotinylated PTX3 with or without unlabeled N_PTX3 or N_PTX3C47S/C49S/C103S for 1 h at 37 °C.

## Source of funding

This work was mainly supported by a grant from Ministero Istruzione, Università e Ricerca to A.S. A.I. would like to acknowledge the Scuola Europea di Medicina Molecolare (SEMM) funding.

## Disclosure statement

The authors R.L. and G.S. are employees of the pharmaceutical company Sigma Tau, which holds patent rights on PTX3 and fertility. This paper does not raise any conflicts of interest.
